# Synergistic Effect of Colistin and Rifampin Against Multidrug Resistant *Acinetobacter baumannii*: A Systematic Review and Meta-Analysis

**DOI:** 10.2174/1874285801711010063

**Published:** 2017-04-28

**Authors:** Maryam Mohammadi, Hatef Khayat, Koroush Sayehmiri, Setareh Soroush, Fatemeh Sayehmiri, Somayeh Delfani, Lidija Bogdanovic, Morovat Taherikalani

**Affiliations:** 1 Clinical Microbiology Research Center, Ilam University of Medical Sciences, Ilam, Iran; 2 Department of Biostatistics, School of Medicine, Ilam University of Medical Sciences, Ilam, Iran; 3 Razi Herbal Medicines Research Center & Department of Microbiology, School of Medicine, Lorestan University of Medical Sciences, Khorramabad, Iran; 4 Proteomics Research Center, Shahid Beheshti University of Medical Sciences, Tehran, Iran; 5 Department of Public Health, University of Naples Federico II, Naples, Italy

**Keywords:** Synergism, Colistin, Rifampin, *A. baumannii*

## Abstract

The existence of infections caused by multidrug resistant (MDR) *Acinetobacter baumannii* is a growing problem because of the difficulty to treat them. We examined the published literature and focused our analysis on the investigation of the synergism of colistin and rifampin against MDR *A. baumannii* isolates *via* systematic review and meta-analysis. A systematic literature search was performed using the following 4 databases (PubMed, Scopus, EMBASE and ISI Web of Sciences). The related articles were evaluated during the period from December 2014 to January 2015. Information based on resistance and sensitivity to antibiotics, the minimum inhibitory concentration and the effects of two antibiotics on each other including synergism, antagonism, relative synergism and additive antagonism were extracted. A meta-analysis of 17 studies including 448 samples was brought into process and 2% (95% CI 0-4%) and 72% (95% CI 56-89%) resistance to colistin and rifampin were observed, respectively. 42% of all isolates showed MIC = 4 µg/ml (95% CI 14-69%) to rifampin and 30% MIC= 2 µg/ml to colistin (95% CI 3.8-78%). MIC_50_ and MIC_90_ for both rifampin and colistin were 2 µg/ml and 4 µg/ml, respectively. 63% of the strains demonstrated synergy (95% CI 37-90%), 7% were highlighted as relative synergism (95% CI 0.0- 13%), 3% showed an additive effect (95% CI -0.0-7%) and 14% were indifferent (95% CI 6-23%). The antagonistic effect was not observed in this combination. Synergy rates of time-kill assay in rifampin and colistin combinations were generally higher than those of check bored microdilution and E-test method. The results demonstrated that the combination therapy could be more useful when compared to monotherapy and that this strategy might reduce the resistance rate to rifampin in MDR *A. baumannii* isolates.

## INTRODUCTION

*Acinetobacter baumannii* is considered as an important cause of healthcare associated infections, particularly in the intensive care units (ICU), and many reports have indicated that antibiotic misuse leads to appearance of resistance strains [1, 2]. So, a lot of efforts have been contributed to find out a solution in order to treat theses nosocomial pathogen, one of which is combined therapy [3, 4]. The combinations of two antibiotics have shown different effects on each other and in many cases the effect is synergistic or strengthening but in some cases, antagonism is observed [5, 6]. combination therapy is usually used in life-threatening infections, to cover a wide spectrum broad against all potential pathogens, to induce synergistic effects of the combination against a specific pathogen to prevent resistance emergence, or to combat a polymicrobial infection not easily treated with a single medication. In combination therapy the balance in its potential disadvantages, including, the possible increase in side effects, superinfection, antagonistic activity and increase in cost should be considered. Combination therapy might also be used to prevent the side effects of a specific drug [7].

Rifampin is an antibiotic which is frequently used with other antibiotics, and the results have shown synergy with colistin; however, the result of this combination is dependent on various conditions such as the rifampin’s MICs or the methods used. For example, the enhancement effects have not been observed in the strains with MIC higher than 256µg/ml [8, 9]. We focused on the studies that examined the combination effects of rifampin and colistin against *Acinetobacter in vitro*. The aim of this study was to obtain the information about the activity of rifampin and colistin and their effects on *A. baumannii* isolates through a review of existing literature and data analysis.

## MATERIALS AND METHOD

### Data Source and Criteria for Selecting Articles

**Chart (1):**
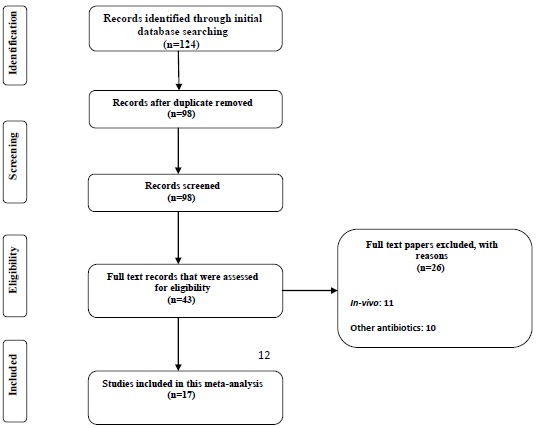
*In-vitro* susceptibility rate of colistin alone against *A. baumannii*. Meta-analysis show that 2% of the isolates were resistant to colistin alone.

In the period from December 2014 to January 2015 the detailed databases (PubMed, Scopus, EMBASE and ISI Web of Sciences) search was performed. The inclusion criteria for meta-analysis were the studies that had examined the interactions of the two antibiotics (rifampin and colistin) and all *in vitro* combination therapies (Checkerboard, Time-kill). The posters printed (ECCMID) from 2007 to 2014 were also checked (the ISI Web of Science web site) (flow chart. **Chart 1**. The key words used were " *A. baumannii* + colistin, *A. baumannii* + rifampicin, " *A. baumannii* + colistimethate " and *A. baumannii* + colistin and rifampicin ". In order to avoid bias, the search was performed by 2 independent researchers.

### Interaction Analysis and Data

The following information-researcher’s name, year, country, number of strains, strain’s resistance and sensitivity to rifampin and colistin, MIC and type of interaction including additive, partially synergism, synergism and antagonism and applied methods were extracted.

The interaction between two antibiotics was evaluated by two methods (Checkerboard, Time-kill) and the initial results of the *in vitro* effects on the bacteria were based on inhibition or killing. In the time-kill method the synergism was defined as the reduction by the amount of log 2 of CFU/ml of the bacteria in the presence of antibiotics’ combination compared to the single state and as antagonism, if it showed an increase. For the checkerboard method the index FICI was applied and the amount of it was obtained by the sum of the two drugs MIC divided by each drugs individual MIC.

FICI=(MIC of drug A in combinationMIC of drug A alone)+(MIC of drug B in combinationMIC of drug B alone)

The results were interpreted according to the following crieteria: FICI≤0.5, synergistic; 0.5 < FICI < 1, partially synergistic; FICI = 1, additive; 1> FICI≤4, indifferent; and FICI > 4, antagonistic.

### Data Analysis

The heterogeneity between studies was assessed by Chi-squared test with significance level of 0.05. and *I* ^2^ test. Heterogeneity was considered as *I* ^2^ 50%. The random effects model was applied to combine the studies' results. The data analysis was performed by STATA software version 11.1.

## RESULT

A total of 104 articles were found in the initial search. The titles and abstracts were reviewed and after the exclusion of unrelated articles, 17 studies entered the meta-analysis. The included articles were 1 poster from ECCMID, 1 short communication, 1 letter to editor, 1 brief report and 13 original articles (Table **1**) [10 - 22]. The sensitivity of 448 strains was analyzed and 2% and 72% resistance to colistin and rifampin when administrated individually were observed, respectively. We also analyzed the abundance of the strains` MIC comparing these two antibiotics.

The range of MIC for rifampin and colistin was 0.25 μg/ml to 64 μg/ml and 0.25 μg/ml to 16 μg/ml, respectively. The majority of strains (42%) demonstrated MIC= 4 and 30% had MIC= 2 for rifampin and colistin, respectively. The interaction between these two antibiotics was analyzed by the Time-kill method (11 studies) and the Checkerboard method (6 studies). The results demonstrated synergy in 63%, while, partial synergy, additive effect and no effect were present in 7, 3 and 14% of the cases, respectively. No combinations were antagonistic. Results and its details were shown in Figs. (**1**-**6**).

## DISCUSSION

Recent increase of the healthcare associated infections caused by MDR strains of *A. baumannii* is becoming a serious problem. The combination antibiotic therapy is proven to reduce the resistance and increase the efficiency of antibiotics. Two antibiotics that are commonly used in combination therapy are colistin and rifampin. There is no reliable information about the amount of resistance to these two antibiotics, especially to rifampin, and resistance pattern varies from hospital to hospital. Therefore, we analyzed the amounts of synergism and resistance to colistin and rifampin and their relationship with the MIC. After analyzing 448 strains, it was identified that 72% of the strains were resistant towards rifampin. Interestingly, in 4 studies the amount of resistance to rifampin was 100%. In one of the studies no synergy was observed, while in another study in 50% of the strains, the two antibiotics had no effect on each other. This shows that the relationship between MIC and the interactions of the two antibiotics are very important [5]. On the contrary, another study showed that 49% of the strains were sensitive to rifampin and in 100% of the cases synergism was observed. It seems that higher MIC and the consequent increase of the resistance to the antibiotic, caused the decrease of the antibiotic`s efficiency and amount of synergism. It is notable that in one of the studies, 6% of the XDR strains were antagonistic towards antibiotics and no synergism was observed in this study. In this study, in 88% of the strains the combination of the two antibiotics was ineffective (13). These results suggested that the increase in resistance to rifampin reduces synergism. The synergic mechanism of the two antibiotics is based on colistin’s effect to the outer membrane of gram-negative bacteria which causes increasing penetration of rifampin into the bacterial cell. One of the mechanisms of resistance to rifampin is *rpo* gene mutation, which causes high-level of resistance, with the concentration of bacterial MIC reaching 256 μg/ml. In Zarrilli and colleagues study the synergy of two antibiotics on the strains of *Acinetobacter* was examined and in one of the strains, where the synergism was not found, the study had identified mutations in the *rpo* gene with MIC higher than 512 μg/ml [9-18]. The resistance to colistin, observed in 3 studies, was found in 2% of the strains. In one of the studies that took place in Taiwan, from the total of 134 MDR strains of *A. baumannii*, 6 strains (10.2%) were resistant to colistin. The same strains were destroyed in the synergistic test [19]. In a study conducted in China, from the 25 strains of XDR *A. baumannii* 3 strains were resistant to colistin. The synergism test results showed 56%, 36% and 0.8% relative to synergism, additive effect and indifference to the two antibiotics, respectively [20-22]. These cases demonstrated the importance of combination therapy against MDR strains, and that the use of combination of two antibiotics can also control the strains resistant to colistin. The mechanism of synergy between two antibiotics in strains resistant to colistin is unknown and it needs further studies. The limitations of the current study were the inclusion of some studies that only mentioned synergism and not referring to interactions such as indifferent or antagonism. Also, some of the studies did not mention the strains’ MIC fully and accurately, however, we tried to do calculation and analysis with less bias possible. In addition, in some cases the breakpoint for rifampin in *A. baumannii* was not defined, and in some articles the break point of *Staphylococcus aureus* for rifampin was used. In this study, the different effects of two antibiotics and their relationship with MIC were conducted in a systematic review method. As a result, in 63% of the strains synergy was observed. This study investigated the interaction of two antibiotics and recommend the use of combination therapy in the treatment of infection caused by *A. baumannii,* especially by MDR strains. The situation in these two phases is not the same, due to different pharmacodynamic and pharmacokinetic effects of different drugs in the host and drug concentration at the site of infection. However, for further simulation of the 2-phase in the time-kill method we used 6 mg/l for colistin and 5mg/l for rifampin, which are suitable concentrations for the human body. Greater use of this method seems to be due to the similarity in the concentration. In addition, the results of a study indicated that the resistance to rifampin occurs after 48 to 72 hours [11]. By increasing the time of the Time-Kill test we can compare the interaction of the two antibiotics better. *In vitro* studies were not able to evaluate the toxicity of the drug, this issue being more important for colistin. The other problem of *in vitro* studies is the lack of generalization and its use in treatment. In detailed *in vitro* studies by examining the MIC of antibiotics in 2 phases (single and in combination), the resistance was evaluated, but in the body phase, we were not able to check the resistance. Thus, more research should be done to fix these problems. The combination therapy has several advantages and one of them is that by using a combination of 2 antibiotics the concentration of each antibiotic can be reduced. This issue, as mentioned above, is more important for colistin. On the other hand, in this study, most of the strains showed MIC=4 for rifampin (42%) and MIC=2 for colistin (30%). In the observed studies, MIC of each antibiotic was calculated individually and then for the combination of two antibiotics the sub inhibitory concentration or a concentration lower than MIC was applied. We suggest an experimental basis to combine two antibiotics with a concentration of 2ug/ml for rifampin and 1 ug/ml for colistin, based on our study results. However, depending on the resistance of the strains (XDR or MDR) these concentrations could be different. It is recommended to use an accurate test such as E-test or micro dilution to measure the MIC of each of the 2 antibiotics first, because the synergy depends on rifampin. Examination of the strains’ MIC can help predict the effect of the two antibiotics on each other.

## CONCLUSION

In this study, based on a systematic review and analysis of the existing studies we have shown that rifampin and colistin had a significant synergy in the in-vitro phase.

## Figures and Tables

**Fig. (1):**
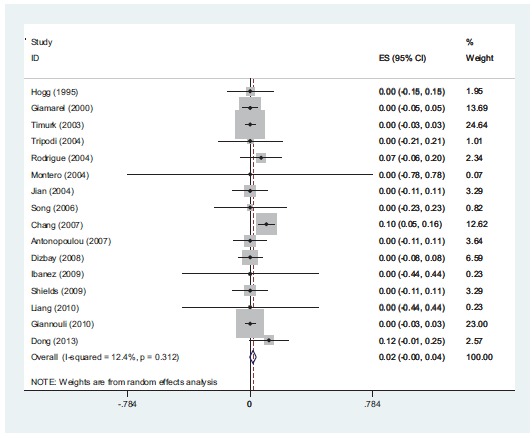
*In-vitro* susceptibility rate of colistin alone against *A. baumannii*. Meta-analysis show that 2% of the isolates were resistant to colistin alone.

**Fig (2):**
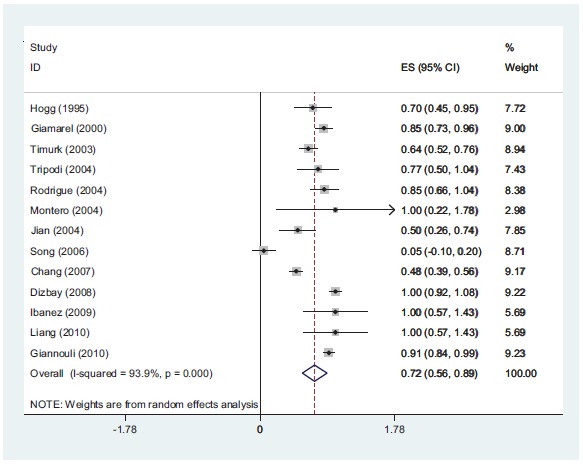
*In-vitro* susceptibility rate of rifampin alone against *A. baumannii*. meta-analysis show that 72% of the isolates were resistant to rifampin when it uses alone.

**Fig (3):**
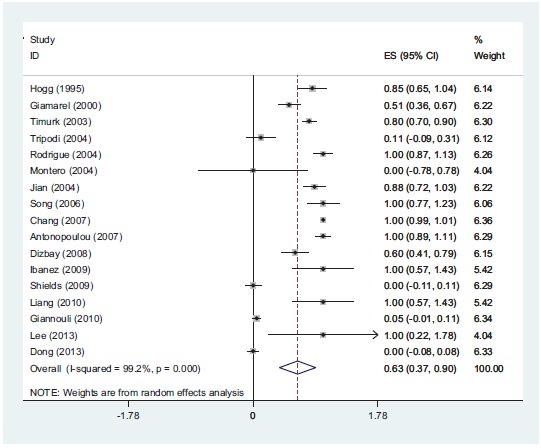
*In-vitro* synergistic rate of colistin with rifampin against *A. baumannii*. according to meta-analysis 63% of the isolates show synergy.

**Fig (4):**
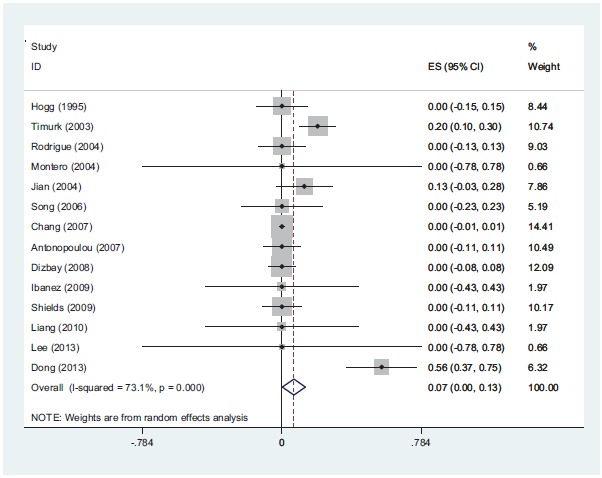
*In-vitro* partially synergistic rate of colistin with rifampin against *A. baumannii* is 7%.

**Fig (5):**
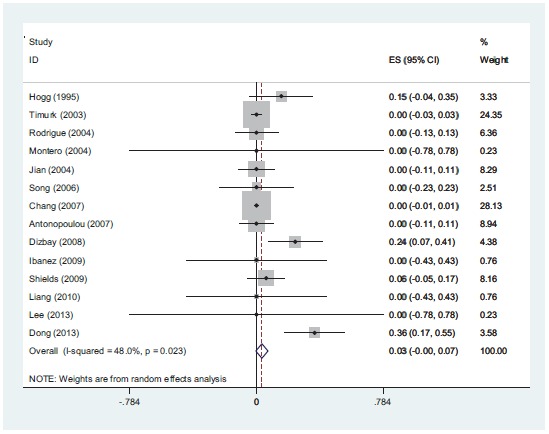
*In-vitro* additive rate of colistin with rifampin against *A. baumannii* is 3%.

**Fig (6):**
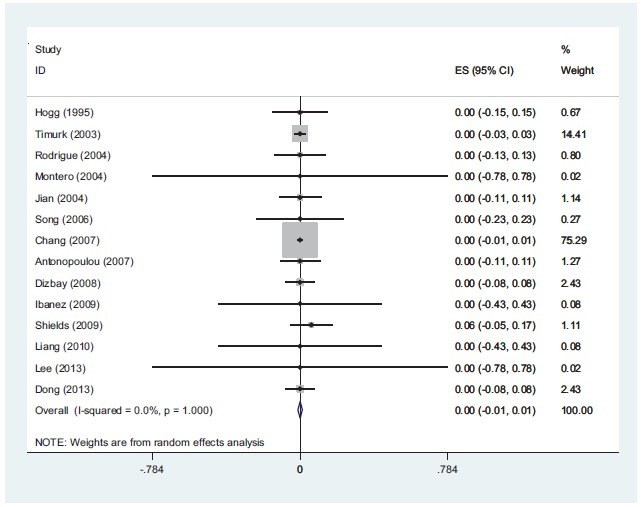
*In-vitro* antagonism rate of colistin with rifampin against *A. baumannii*. The result show that there is not antagonism between colistin and rifampin against *A. baumannii* isolates.

**Table 1:** Characteristics of the Studies Included in Meta-analysis.

**Reference**	**Country**	**Published year**	**No. of isolates**	**Susceptibility** **test**	**Synergy method(s)**
**Timurk [****10****]**	**Turkey**	**2005**	**60**	**Agar dilution**	**Checkerboard**
**Tripodi [****6****]**	**Italy**	**2007**	**9**	**Micro dilution**	**Time-kill**
**Ibanez [****11****]**	**Spain**	**2010**	**4**	**Agar dilution**	**Time-kill**
**Lee [****12****]**	**USA**	**2013**	**2**	**Micro dilution**	**Time-kill**
**Shields [****13****]**	**USA**	**2010**	**17**	**E-test**	**Checkerboard**
**Rodriguez [****14****]**	**Argentina**	**2010**	**14**	**Agar dilution**	**Time-kill**
**Giamarel [****8****]**	**Greece**	**2001**	**39**	**Micro dilution**	**Time-kill**
**Song [****15****]**	**South Korea**	**2007**	**8**	**Micro dilution**	**Time-kill**
**Montero [****5****]**	**Spain**	**2004**	**2**	**Micro dilution**	**Time-kill**
**Hogg [****16****]**	**England**	**1998**	**13**	**Micro dilution**	**Checkerboard**
**Liang [****17****]**	**China**	**2011**	**4**	**Micro dilution**	**Time-kill**
**Dizbay [****18****]**	**Turkey**	**2009**	**25**	**E-test**	**Checkerboard**
**Chang [****19****]**	**Taiwan**	**2010**	**134**	**Micro dilution**	**Checkerboard**
**Dong [****20****]**	**China.**	**2014**	**25**	**Micro dilution**	**Checkerboard**
**Jian [****21****]**	**Australia**	**2007**	**17**	**Micro dilution**	**Checkerboard**
**Giannouli [****9****]**	**Italy**	**2011**	**57**	**Micro dilution**	**Time-kill**
**Antonopoulou [****22****]**	**Greece**	**2007**	**18**	**Micro dilution**	**Time-kill**
